# Cutaneous drug reaction with intravenous ceftriaxone

**DOI:** 10.4103/0253-7613.59933

**Published:** 2009-12

**Authors:** Inderpal Kaur, Jatinder Singh

**Affiliations:** Department of Pharmacology, Government Medical College, Amritsar, India; 1 Department of Surgery, Government Medical College, Amritsar, India

## Introduction

Fixed drug eruption (FDE) is a unique pattern of cutaneous drug reaction, characterized by skin lesions that recur at the same site or sites each time the drug is administered. Acute lesions appear as round or oval, sharply marginated erythmatous plaques that sometimes develop central bullae. The lesions are usually found on the lips and genitalia, although any skin or mucosal surface may be involved.[1,2] The eruption usually occurs within hours of administration of the offending agent and resolves spontaneously without scarring after few weeks of onset, usually with residual post-inflammatory pigmentation. The most frequently implicated drugs are sulfonamides, tetracyclines, salicylates and barbiturates.[3]

## Case Report

A 32-year -old female was admitted in the hospital with severe diffuse pain in the abdomen radiating to the back. Laboratory investigations were performed to rule out the possible causes of pain of the abdomen. The ultrasound report revealed presence of hemorrhagic cysts in both the ovaries along with umbilical hernia. Bilateral salpingo-oopherectomy and hernia repair was advised to the patient. She was thoroughly examined and investigated prior to the operation. Pre-operative laboratory test reports were within normal limits, except for hemoglobin, which was 8.5 gm%. Drug history of the patient did not reveal any allergy or hypersensitivity reaction. Perioperatively, prior to anesthesia, the patient was administered 1 g IV ceftriaxone. Surgery was performed under spinal anesthesia and the outcome was uneventful. On the second day of operation, she complained of burning discomfort and itching on the under surface of the left thigh. On examination, an oval reddish patch with a small vesicle of approximately 6 cm ± 2 cm in size was observed. A similar patch with lesser severity was also seen at the parallel site on the right thigh. By the time of examination, the patient had received three doses of ceftriaxone (1 g each) intravenously. Ceftriaxone was stopped and the patient was administered 20 mg of prednisolone and 10 mg of cetrizine. Soframycin and betamethasone ointments were prescribed twice daily for 5 days and cetrizine was continued. The lesions disappeared in 1 week. Concurrent administered medicines, i.e. diclofenac (1 tab p.o., t.d.s.) and injection gentamicin (80 mg im, b.i.d.) were continued for 7 days till the removal of stitches.

## Discussion

Adverse drug reactions (ADRs) are a major hazard of modern medicine. Cutaneous ADRs are an important clinical entity that can endanger the life of the patient. Cephalosporins can induce severe or life-threatening IgE-mediated reactions in some individuals.[4] Causal relationship between the drug and the reaction is assessed depending on the lag period between the start of the drug and the appearance of the reaction, responses to de-challenge and re-challenge tests and the data available regarding the drug. In the present case report, the patient presented with FDE after intravenous administration of ceftriaxone. The exact timing of appearance of symptoms could not be noted by the patient. This was because of the effect of spinal anesthesia, which obviated the cardinal symptoms of itching and burning. The patient showed a positive response to the dechallange test. Previously, a single case of FDE with cephalosporins had been reported by Ozkaya.[5] According to the WHO causality definitions,[6] this ADR is categorized as a certain reaction to the drug. Lack of cross-sensitivity between most of the cephalosporins[7] is suggested in this case, which showed hypersensitivity to injectable ceftriaxone and no ADR to oral cefixime. This suggests that the beta lactam ring may not be responsible for hypersensitivity because the side chain-specific antibodies predominate in the immune response to cephalosporins.[8] Although documentation of ADRs can significantly contribute to quality assurance in drug therapy in routine clinical practice, these remain largely unrecorded. The manufacturers of injectable ceftriaxone should incorporate the possibility of a FDE as an ADR in their package inserts and other drug information documents.
